# *Mycobacterium ulcerans* low infectious dose and mechanical transmission support insect bites and puncturing injuries in the spread of Buruli ulcer

**DOI:** 10.1371/journal.pntd.0005553

**Published:** 2017-04-14

**Authors:** John R. Wallace, Kirstie M. Mangas, Jessica L. Porter, Renee Marcsisin, Sacha J. Pidot, Brian Howden, Till F. Omansen, Weiguang Zeng, Jason K. Axford, Paul D. R. Johnson, Timothy P. Stinear

**Affiliations:** 1 Department of Biology, Millersville University, Millersville, PA, United States of America; 2 Department of Microbiology and Immunology, at the Peter Doherty Institute for Infection and Immunity, University of Melbourne, Melbourne, Australia; 3 Department of Internal Medicine, University of Groningen, Groningen, RB, The Netherlands; 4 Pest and Environmental Adaptation Research Group, Bio21 Institute and School of BioSciences, University of Melbourne, Parkville, Vic, Australia; 5 Department of Infectious Diseases, Austin Health, Heidelberg, Victoria, Australia; Johns Hopkins Bloomberg School of Public Health, UNITED STATES

## Abstract

Addressing the transmission enigma of the neglected disease Buruli ulcer (BU) is a World Health Organization priority. In Australia, we have observed an association between mosquitoes harboring the causative agent, *Mycobacterium ulcerans*, and BU. Here we tested a contaminated skin model of BU transmission by dipping the tails from healthy mice in cultures of the causative agent, *Mycobacterium ulcerans*. Tails were exposed to mosquito (*Aedes notoscriptus* and *Aedes aegypti*) blood feeding or punctured with sterile needles. Two of 12 of mice with *M*. *ulcerans* contaminated tails exposed to feeding *A*. *notoscriptus* mosquitoes developed BU. There were no mice exposed to *A*. *aegypti* that developed BU. Eighty-eight percent of mice (21/24) subjected to contaminated tail needle puncture developed BU. Mouse tails coated only in bacteria did not develop disease. A median incubation time of 12 weeks, consistent with data from human infections, was noted. We then specifically tested the *M*. *ulcerans* infectious dose-50 (ID50) in this contaminated skin surface infection model with needle puncture and observed an ID50 of 2.6 colony-forming units. We have uncovered a biologically plausible mechanical transmission mode of BU via natural or anthropogenic skin punctures.

## Author summary

Buruli ulcer is a neglected tropical disease caused by infection with *Mycobacterium ulcerans*. Unfortunately, how people contract this disease is not well understood. Here we show for the first time using experimental infections in mice that a very low dose of *M*. *ulcerans* delivered beneath the skin by a minor injury caused by a blood-feeding insect (mosquito) or an experimental needle puncture is sufficient to cause Buruli ulcer. This research provides important laboratory evidence to advance our understanding of Buruli ulcer disease transmission.

## Introduction

Among the 17 neglected tropical diseases the World Health Organization (WHO) has targeted for control and elimination, only Leprosy and Buruli ulcer (BU) have unknown modes of transmission [1]. The search to understand how humans contract BU spans more than 70 years since the causative agent, *Mycobacterium ulcerans*, was first identified [2]. There are persistent and emerging foci of BU cases across the world, in particular Africa and Australia [3]. BU is characterized by necrotizing skin lesions, caused by localized proliferation of *M*. *ulcerans* in subcutaneous tissue. BU is rarely fatal, but untreated infections leave patients with significant disfigurement and disability, with damaging personal and economic consequences [4, 5]. Researchers have long been struck by the characteristic epidemiology of BU, with cases occurring in highly geographically circumscribed regions (sometimes less than a few square kilometres) and risk factors for infection that include gardening, insect bites and proximity to (but not necessarily contact with) lacustrine/riverine regions [6–14]. Human-to-human spread is considered unlikely [14]. Disease transmission is thought to occur by contact with an environment contaminated with *Mycobacterium ulcerans* but exactly where the pathogen resides and why it appears so geographically restricted have yet to be determined. [15].

*M*. *ulcerans* is very slow growing (doubling time >48 hrs) and this poses a problem for source tracking efforts as it is difficult to isolate the bacteria in pure culture from complex environmental specimens [16]. *M*. *ulcerans* has only once been isolated from a non-clinical source, an aquatic water bug (Gerridae) from Benin [16]. Quantitative PCR targeting *M*. *ulcerans*-specific DNA is the most frequently used technique in surveys of environmental specimens. A comprehensive review of the many field and lab studies that have examined transmission of BU has highlighted the range of organisms from aquatic plants, snails, insects, fish, amphibia, and in Australia certain native marsupials that can serve as potential reservoirs for *M*. *ulcerans* [15, 17–20]. Since the first observation that biting aquatic insects can harbour *M*. *ulcerans* [21], studies of BU transmission have largely focused on the potential for insects to biologically vector *M*. *ulcerans* implying that *M*. *ulcerans* undergoes a propagative or reproductive mode of development in an insect [22–26]. Several case-control studies, including from both Australia and Africa have suggested insects may play a role in transmission [10, 11]. For example, in southeastern Australia, we noted Buruli lesions on exposed areas likely to attract biting insects, some patients with every brief exposure times to endemic areas [27, 28] and in 2004 we began a study that identified *M*. *ulcerans* DNA associated with mosquitoes captured in endemic areas [22]. However, there is no compelling experimental evidence for single-mode biological transmission of *M*. *ulcerans* via insect vectors.

## Materials and methods

### Ethics statement

The animal ethics committee (AEC) of the University of Melbourne approved all animal experiments under approval number AEC: 1312775.2, in accordance with the National Health and Medical Research Council Australian code for the care and use of animals for scientific purposes 8th edition (2013).

### Bacterial isolates and culture conditions

*M*. *ulcerans* strain JKD8049 and bioluminescent *M*. *ulcerans* JKD8049 (harbouring plasmid pMV306 *hsp*:*lux*G13) [29, 30] were cultured in 7H9 broth or Middlebrook 7H10 agar, containing 10% oleic-albumin-dextrose-catalase growth supplement (Middlebrook, Becton Dickinson, Sparks, MD, USA) and 0.5% glycerol (v/v) at 30°C. Colony counts from bacterial cultures or tissue specimens were performed using spot plating. Five x 3μl volumes of serial 10-fold dilutions (10^−1^ to 10^−5^) of a culture or tissue preparation were spotted onto 7H10 agar plates with a 5x5 grid marked. The spots were allowed to dry, the plates loosely wrapped in plastic bags and then incubated as above for 10 weeks before counting colonies. Data analysis was performed using GraphPad Prism v 7.0a. All culture extracts were screened by LC-MS for the presence of mycolactones as previously described to ensure bacteria used in transmission experiments remained fully virulent [31].

### Experimental animals

BALB/c mice were purchased from ARC (Canning Vale, Australia) and housed in individual ventilated cages. Upon arrival, animals were acclimatizing for 5 days. Food and water were given *ad libitum*.

### Aedes notoscriptus and Aedes aegypti rearing

Wild caught mosquitoes were sourced from around Cairns, Queensland, Australia. *A*. *notoscriptus* and *A*. *aegypti* colonies were reared in a Physical Containment Level 2 (PC2) laboratory environment at 26°C using previously described methods, with the addition of brown paper used as the oviposition substrate for *A*. *notoscriptus* [32]. Females were aged for at least one week prior to blood feeding ensure maturity, during which they were mated (seminal accessory fluid is required to stimulate searching and feeding behavior in females). The adults were provided with access to a 10% sucrose solution, which was withdrawn 24h prior to a blood feeding experiment.

### Mosquito-mouse transmission experiments

Six-week old female BALB/c mice were anaesthetized and their tails coated in a thin film of *M*. *ulcerans* wild type strain JKD8049 by dipping the tails in a Petri dish containing 20mL of bacterial culture (concentration ~10^6^ CFU/mL) (Table 1). Tails were allowed to air-dry for 5 minutes after dipping. The tail only was then exposed to a 200mm x 200mm x 200mm cage of 20 adult female mosquitoes for a period of 15 minutes. Twenty mosquitoes were used per feeding bout to minimize stress on the mice. The number of insects biting each mouse was recorded over the exposure period by continuous observation. Tails were not wiped or disinfected post-biting. Mice were then observed weekly for up to six months for signs of tail lesions. Sterile needle stick (25G or 30G needle) and no-trauma were used as controls. An additional control consisted of tails dipped in sterile culture broth only and subjected to mosquito biting or sterile needle stick (See Table 1 for experiment design). The concentration of bacteria used was calculated by dilution plating as described above. Contingency tables and Fisher’s exact test were used to test if instances of mosquito-associated or needle stick-associated disease transmission were significantly different to no trauma (GraphPad Prism v 7.0a).

**Table 1:** Summary of transmission experiments.

Trauma source	Number of mice	Mouse tail coated	Number of mice bitten	Number of mice developing BU^3^	Estimated dose (CFU)
***Experiment 1*:**					
*Aedes notoscriptus*	12 (4/mosquito cage^1^)	*M*. *ulcerans* (4.1x 10^6^ CFU/mL)	6	2	21
*Aedes notoscriptus*	4 (1/mosquito cage)	Media-only	2	0	-
Sterile needle (25G)	5	*M*. *ulcerans* (4.1x 10^6^ CFU/mL)	-	4	55
None	6	*M*. *ulcerans* (4.1x 10^6^ CFU/mL)	-	0	-
***Experiment 2*:**					
*Aedes aegypti*	5 (1/mosquito cage)	*M*. *ulcerans* (1.83 x 10^6^ CFU/mL)	5^2^	0	9
*Aedes aegypti*	3 (1/mosquito cage)	Media-only	2	0	-
Sterile needle (25G)	5	*M*. *ulcerans* (1.83 x 10^6^ CFU/mL)	-	4	40
None	5	*M*. *ulcerans* (1.83 x 10^6^ CFU/mL)	-	0	-
***Experiment 3*:**					
Sterile needle (30G)	14	*M*. *ulcerans* (3.9 x 10^6^ CFU/mL)	-	13	14
None	8	*M*. *ulcerans* (3.9 x 10^6^ CFU/mL)	-	0	-

Notes

^1^There were 20 adult female mosquitoes per cage

^2^Multiple bites per mouse with 2 mice receiving 3 bites and 1 mouse receiving 2 bites. All other mice received only one bite.

^3^IS2404 qPCR results between mosquito and needle puncture experiments were not significantly different (average Ct = 20.22, range 16.52–33.5).

### Infectious dose calculations and infectious dose 50 experiment

**Fig 1:**
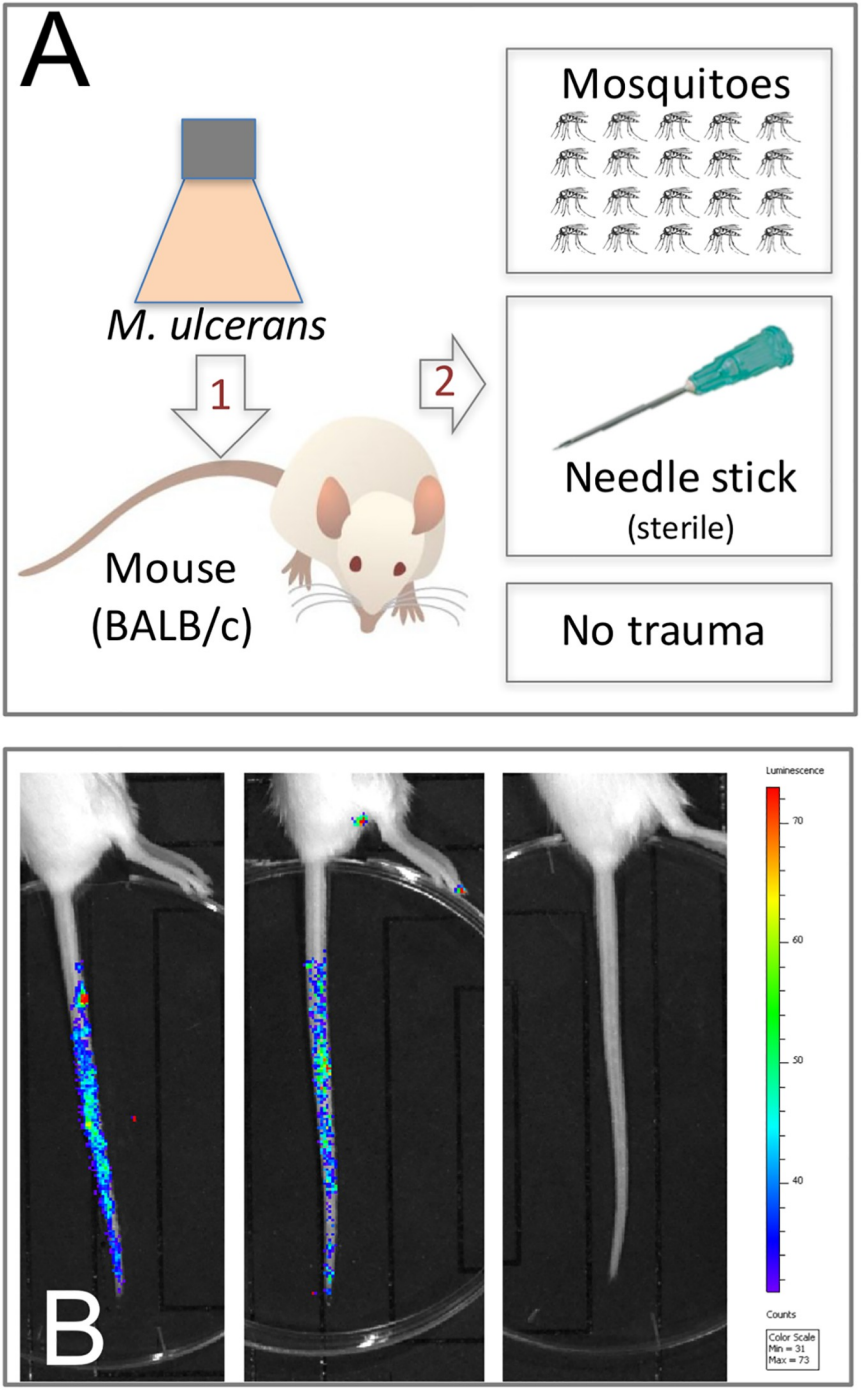
Schematic representations of the two BU transmission models tested in this study. (A) Model-1 tests transmission of *M*. *ulcerans* present on a skin surface following a puncturing injury created by mosquito blood-feeding or needle stick. (B) Visualization of bioluminescent *M*. *ulcerans* JKD8049 (harbouring plasmid pMV306 *hsp*:*lux*G13) [29, 30] on the mouse-tail in model-1, showing the distribution of bacteria immediately after coating for two mice, versus an uncoated animal. *M*. *ulcerans* culture concentration used for tail coating was 8.3x10^5^ CFU/mL.

To estimate the infectious dose, we first measured the surface area of five dissected naïve mouse-tails to obtain an average surface area (493.3 ± 41.1 mm^2^). Using ten naïve mouse-tails and a precision balance, we then calculated the average volume of *M*. *ulcerans* 7H9 Middlebrook culture adhering to the tail surface (32.4 ± 4.2 μL), the concentration of bacteria in the cultures used, and the surface area of the tips of 25G and 30G needles used to deliver the puncture wounds (0.207 mm^2^ and 0.056 mm^2^ respectively). These parameters were then used to calculate the infectious dose, assuming the bacteria were evenly distributed over the tail surface (Fig 1B). A standard protocol to calculate an ID50 was followed. Four-week old female BALB/c mice were anaesthetized and their tails coated as described above for the transmission experiments with 10-fold dilutions of bioluminescent *M*. *ulcerans* JKD8049 from 10^6^ to 10 CFU/mL in each Petri dish, using five mice per dilution, followed by a single needle stick puncture with a 25G needle. The mice were monitored using a Lumina XRMS Series III In Vitro Imaging System (IVIS) (Perkin Elmer). Images were captured with the following settings: binning of 4 (medium), Field of View (FOV) of 24, Relative aperture at f1.2 and exposure time of 180s. Bioluminescence was calculated using Living Image software V4.1. The number of mice per dilution developing BU up to six-months was recorded. Mice were observed up to six-months. The data were plotted and a standard curve was fitted using least squares non-linear regression. The ID50 and 95% confidence intervals were interpolated with reference to the standard curve using GraphPad Prism v 7.0a.

### Real time quantitative PCR

For each mosquito that blood-fed DNA was individually extracted from the dissected head, abdomen and legs of each insect using the MoBio Powersoil DNA extraction kit following manufacturer’s instructions (MoBio Laboratories Inc., Carlsbad CA USA). The insects were held by the wings and each body portion (legs, abdomen, head) individually and separately removed with sterile, fine forceps to avoid cross contamination during dissections. Body parts were stored individually (legs were pooled per insect) in sterile 1.5mL tubes at -20°C until DNA extraction. DNA was similarly extracted from mouse tissue. Procedural extraction control blanks (sterile water) were included at a frequency of 10% to monitor potential PCR contamination, in addition to no-template negative controls. IS*2404* quantitative PCR (qPCR) was performed as described [33]. IS*2404* cycle threshold (Ct) values were converted to genome equivalents (GE) to estimate bacterial load within a sample by reference to a standard curve (r^2^ = 0.9312, y = [-3.000Ln(x)+39.33]*Z, where y = Ct and x = amount of DNA [fg] and Z = the dilution factor]), calculated using dilutions of genomic DNA from *M*. *ulcerans* strain JKD8049, quantified using a fluorimeter (Qubit, Invitrogen) [33].

### Preparation of mouse tissue for analysis

At the end of the experimental period or when a clinical end-point was reached mice were humanely killed. The region of a mouse-tail spanning a likely lesion was cut into three equal sections for histology, qPCR and CFU counts. Individual tail pieces for CFU counts were weighed and placed into sterile 2ml screw capped tube containing 0.5g of large glass beads and 600μl of sterile 1x PBS. Tissues were homogenized using four rounds of 2x 30second pulses in a high-speed tissue-disruptor at 6500 rpm, with tubes placed on ice for 5 minutes between each round. A 300μl volume of this homogenate was decontaminated with 300μl of 2% NaOH (v/v) and incubated at room temperature for 15 minutes. The preparation was neutralized drop-wise with a 10% solution of orthophosphoric acid (v/v) with added bromophenol blue until the solution changed from blue to clear. The mixtures were diluted in PBS and CFUs determined by spot plating as described above.

### Histology

Sections of mouse-tails were fixed in 10% (w/v) neutral-buffered-formalin and imbedded in paraffin. Each mouse-tail was sectioned transversely (4μm thick) and subjected to Ziehl-Neelson and hematoxylin/eosin staining. The fixed and stained tissue sections were examined by light microscopy.

## Results

### M. ulcerans is efficiently transmitted to a mammalian host by mechanical means

**Fig 2:**
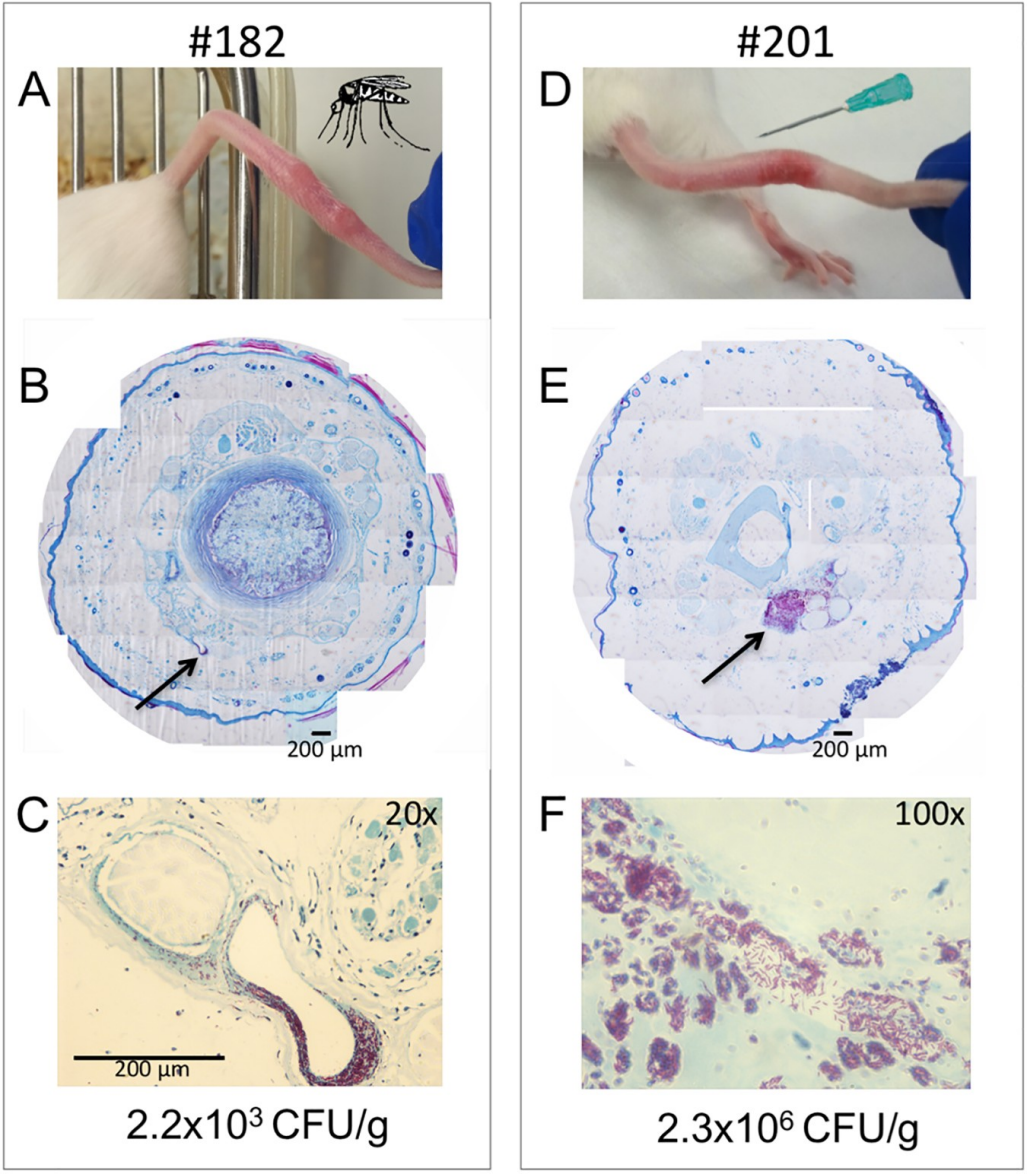
Mechanical transmission of *M*. *ulcerans*. (A) An example of the development of Buruli ulcer following mosquito blood-feeding through a skin surface (mouse-tail) contaminated with *M*. *ulcerans*. (B) Composite histological cross-section with Ziehl–Neelsen staining through the infected tail showing the focus of acid-fast bacilli (arrow) within the subcutaneous tissue. (C) Higher magnification view of the focus of infection, with the yield of viable M. ulcerans obtained from the infected tissue. Panels (D)–(F) show the same analyses as for the mosquito-bitten mouse #182, but for a mouse developing a lesion following sterile needle-stick puncture through a contaminated skin surface (mouse #201).

In experiment one, we established a murine model of *M*. *ulcerans* transmission that represented a skin surface contaminated with the bacteria and then subjected to a minor penetrating trauma, via either a mosquito bite or needle stick puncture. For this experiment, only *Aedes notoscriptus* mosquitoes were used, a local species previously associated with BU in south east Australia [22]. We first coated the tails of 12 mice with *M*. *ulcerans* then exposed only the tails to *A*. *notoscriptus*. Six of the 12 mice exposed to mosquitoes were bitten once each, and of these six mice, two developed BU lesions (Table 1, Fig 1B, Fig 2A). Histology of these lesions confirmed a subcutaneous focus of AFB, within a zone of necrotic tissue. There was also characteristic epithelial hyperplasia adjacent to the site of infection (Fig 2B and 2C). Material extracted from the lesions was IS*2404* qPCR-positive and culture positive for *M*. *ulcerans* (S1 Table). Mice bitten by mosquitoes but with tails coated only with sterile culture media did not develop lesions (Table 1). In the same experiment, we also subjected five mice to a single needle stick puncture. Each mouse had their tail coated with *M*. *ulcerans* as for the mosquito biting. Four of these five mice developed *M*. *ulcerans* positive lesions (Table 1, Fig 2D), with subcutaneous foci of infection and viable bacteria (Fig 2F). The histology of these lesions was the same as the mice subjected to mosquito blood feeding, however bacterial burden was higher following needle stick puncture (Fig 2C compared with Fig 2F). Six mice with their tails coated with *M*. *ulcerans* but not subjected to a puncturing injury did not develop lesions and remained healthy until the completion of the experiment at six months. This experiment suggested that minor penetrating skin trauma (defined here as a puncture <0.5mm diameter and <2mm deep) to a skin surface contaminated with *M*. *ulcerans* is sufficient to cause infection. It also revealed a means by which mosquitoes could act as mechanical vectors of *M*. *ulcerans*.

In experiment two, using approximately the same dose of bacteria to coat the mouse-tails, we repeated experiment-1 but with *Aedes aegypti*, because of the close association of this mosquito to humans world-wide and their potential to vector pathogens. Despite multiple mosquito bites per mouse in the second experiment compared to the first, none of the five *Aedes aegypti*-exposed mice developed lesions (Table 1). However, as for experiment 1, four of five mice subjected to single, needle stick puncture developed *M*. *ulcerans* positive tail lesions (Table 1). A third needle stick puncture experiment was then conducted, this time using a smaller diameter, 30-gauge needle, to assess the impact of a smaller injury. There were 13/14 mice that developed BU when subjected to a single needle stick puncture through a contaminated skin surface, while eight mice with contaminated skin but no injury did not progress to disease (Table 1). Thus, across the three experiments there were 21/24 mice (88%) with needle stick puncture that developed BU, suggesting that this is an efficient mode of disease transmission (Table 1). Either *A*. *notoscriptus* mosquito bite or needle stick trauma significantly increased the risk of developing BU in our mouse skin surface contamination model (Tables 2 & 3).

**Table 2:** Correspondence between *A*. *notoscriptus* biting and development of BU for the contaminated skin model.

	Developed BU	No infection	Total
**Mosquito blood feed**	2*	4	6
**No blood feed**	0	18	18
**Total**	2	22	24

* Fishers exact test: odds ratio 20.56 (95% CI 0.83–507.9), p<0.05

**Table 3:** Correspondence between needle stick puncture and development of BU for the contaminated skin model.

	Developed BU	No infection	Total
**Needle stick puncture**	21*	3	24
**No needle stick puncture**	0	18	18
**Total**	21	21	42

* Fishers exact test: odds ratio 227.3 (95% CI 11.0–4697), p<0.0001

### Higher M. ulcerans burden on mosquitoes is associated with transmission

**Fig 3:**
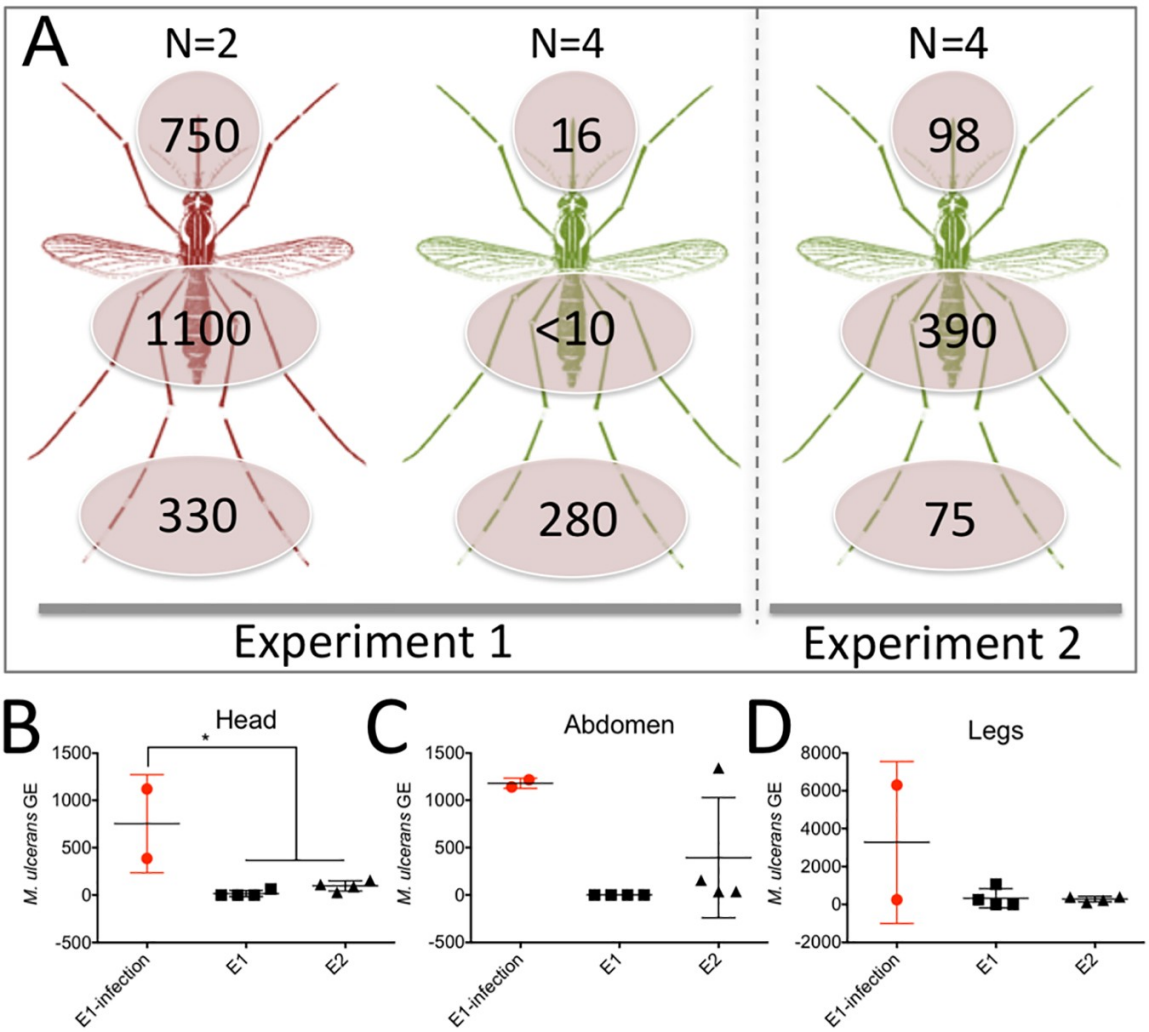
Summary of *M*. *ulcerans* burden on mosquitoes post-feeding on contaminated mouse-tails. (A) Visualization of the mean number of *M*. *ulcerans* detected per dissected mosquito segment, as assessed by IS*2404* qPCR and expressed as genome equivalents (GE). ‘N’ indicates the total number of mosquitoes tested. Red-shaded mosquitoes transmitted *M*. *ulcerans*, leading to mouse-tail lesions. Green-shaded mosquitoes blood-fed on mouse-tails but lesions did not develop. (B, C, D) Plots of the individual qPCR results for each mosquito segment, listed by experiment. Red dots correspond to qPCR bacterial load for mosquitoes that transmitted *M*. *ulcerans* infection. Null hypothesis (no difference in means) was rejected (p<0.05)* (Kruskal-Wallis test). Horizontal bar indicates the mean bacterial load per mosquito and error bars depict standard deviation. The y-axis is GE and x-axis is experiment. The qPCR data for individual insects is contained in S1 Table.

We assessed the likely burden of *M*. *ulcerans* by individual IS2404 qPCR of the head, abdomen and legs for each mosquito that blood fed (Fig 3). A summary of these results is shown in Fig 3A. We noted that the bacterial load (expressed as genome equivalents [GE]) was significantly higher in the mosquito heads associated with mice that developed lesions (p<0.05) (Fig 3B, S1 Table). These data point to a threshold, above which some mosquitoes may become competent mechanical vectors for *M*. *ulcerans* transmission.

### Estimation of incubation period and infectious dose of transmission model-1

**Fig 4:**
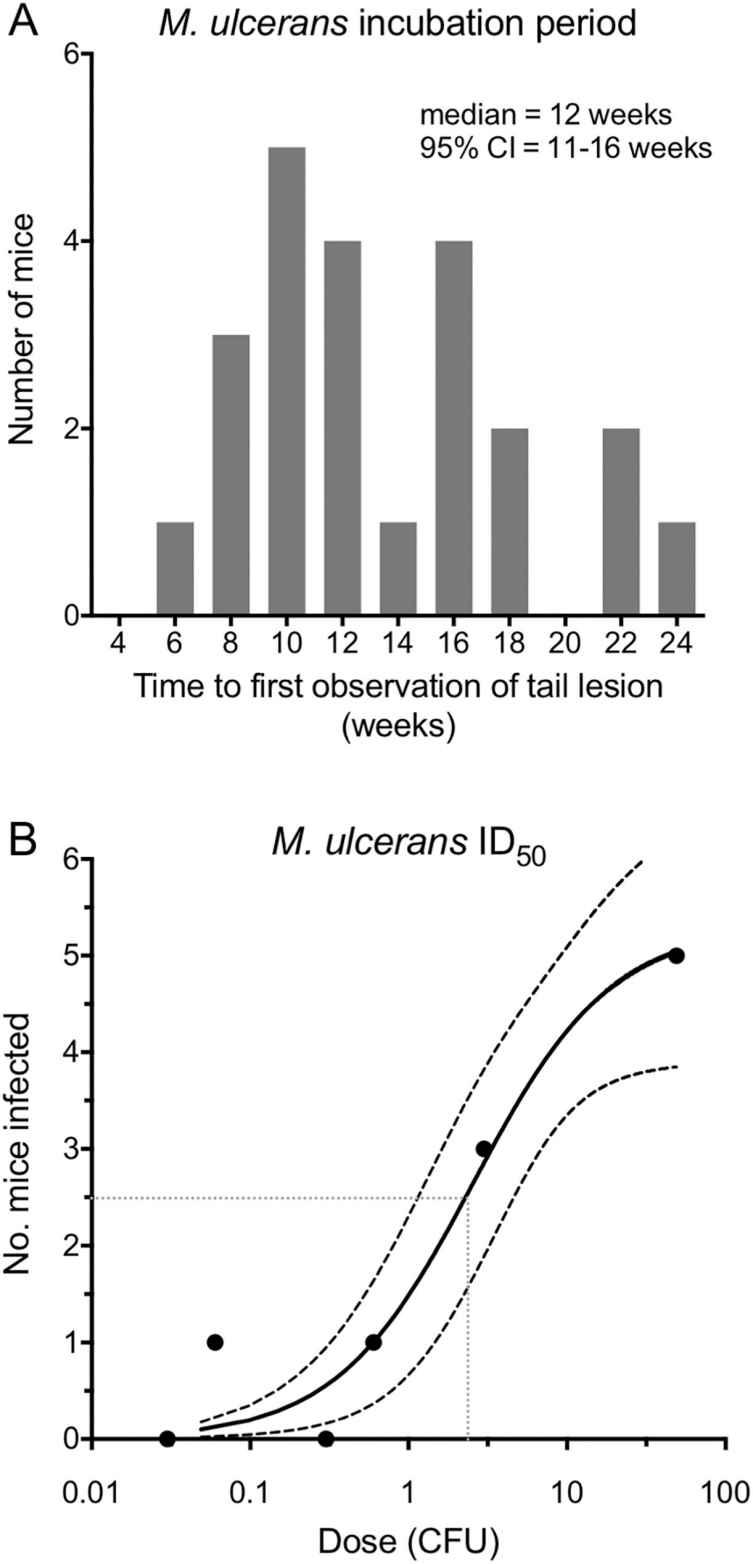
*M*. *ulcerans* incubation period and infectious dose_50_. (A) Incubation period of *M*. *ulcerans* based on the time between sterile-needle puncture of an *M*. *ulcerans* contaminated mouse-tail and first observation of a lesion. (B) Estimated *M*. *ulcerans* ID50 for contaminated skin surface transmission model. Dashed lines indicate 95% confidence intervals. Dotted lines depicts ID50.

Based on the time until a tail lesion was first observed, and when using the highest concentration of bacteria (dose range 9–55 CFU, Table 1) we estimated a median incubation period (IP) of 12 weeks (Fig 4A). This result overlaps with the IP in humans for BU, estimated in different epidemiological studies from 4–10 weeks in Uganda during the 1960s [14] and 4–37 weeks in south east Australia [28]. We then estimated the infectious dose-50 (ID50). We used six different concentrations of *M*. *ulcerans* to coat the tails of mice (n = 5 mice/dilution), subjected each mouse-tail to a single needle stick puncture, and then observed the number of mice for each dilution that developed Buruli ulcer, allowing an ID_50_ estimate of 2.6 CFU (95% CI 1.6–3.6 CFU) (Fig 4B). To our knowledge this is the first estimate of an *M*. *ulcerans* infectious dose and indicates that like *Mycobacterium tuberculosis* and *Mycobacterium leprae*, a small quantity of this slow growing mycobacterium is sufficient to cause disease.

## Discussion

Pathogen transmission by arthropods is generally characterized by either biological transmission, such as malaria [34] or mechanical transmission, where replication or biological transformation of the pathogen within the vector is not necessary for disease spread [35, 36]. Here, we show for the first time an efficient mechanical mode of transmission of *Mycobacterium ulcerans* to a mammalian host that implicates both puncturing injuries and arthropods. In our study, the uninfected host is externally contaminated with *M*. *ulcerans* that is certainly plausible in many areas of the world. We propose that a micro-puncture wound of any sort whether it is by natural means e.g., a thorn, arthropod bite or artificially induced via a human-mediated puncture has the potential to inject *M*. *ulcerans* and generate an ulcer. This research was designed around established frameworks for implicating vectors in disease transmission and provides the necessary causational evidence to help resolve the 80-year mystery on how *M*. *ulcerans* is spread to people [15, 37]. The efficient establishment of BU we have shown here via minor penetrating trauma such as a needle puncture through a contaminated skin surface helps fulfil one of four Barnett Criteria [37]. In vector ecology, mechanical transmission is defined as a non-circulative process involving accidental transport of the pathogen [36]. That is, the pathogen, in some fashion, nonspecifically associates or contaminates the mouthparts (stylet) of an arthropod vector. Insect mechanical transmission of BU implies that if *M*. *ulcerans* were ingested and then egested via regurgitation or salivation, the mechanism would act more like a syringe than a needle [38]. Such a mode of *M*. *ulcerans* disease transmission is supported by previous laboratory studies in which *Naucoris* and Belostmatid water bugs were contaminated via feeding on maggot prey that had been injected with *M*. *ulcerans* or fed naturally on dietary contaminated larval mosquito prey [26, 39].

Our demonstration in the current study of mechanical transmission suggests there are potentially multiple or parallel pathways of *M*. *ulcerans* infection [37]. Examples of bacterial diseases with multiple transmission modes include tularemia, plague and trachoma [40, 41]. Support for our mechanical transmission model also comes from the many field reports over the decades of *M*. *ulcerans* infection following trauma to the skin. Case reports have noted BU following a suite of penetrating injuries ranging from insect bites (ants, scorpions), snake bite, human bite, splinters, gunshot, hypodermic injections of medication and vaccinations [42–44]. Epidemiologists in Uganda during the 1960s and 70s suggested sharp-edged grasses might introduce the bacteria [45]. However, a recent laboratory study established that abrasions of the skin in Guinea pig models and subsequent application of *M*. *ulcerans* was not enough to cause an ulcer, however, this same study established that a subcutaneous injection would cause an ulcer [46]. As a sequel to this study in Guinea pigs, we raised the question of how likely it was that mammalian skin could be sufficiently coated in *M*. *ulcerans* that an injury from natural or anthropogenic sources could lead to infection. Other explanations for the transmission of *M*. *ulcerans* include linkages with human behavior that increase direct contact with human skin and contaminated water [15]. A recent study from Cameroon recorded the persistence of *M*. *ulcerans* over a 24-month period in a waterhole used by villagers (including BU patients) for bathing [47]. A similar study in Ghana documented a 90% positivity rate for MU for water bodies frequented by community members for bathing and washing purposes [48]. Hence, it is reasonable to envisage a scenario where a villager’s skin surface becomes contaminated after bathing in such a water body and is primed for infection if (i) the concentration of bacteria is sufficiently high, and (ii) an inoculating event occurs. Whereas, in Australia, earlier studies have shown that *M*. *ulcerans* contamination of possum feces in and around the gardens of BU patients might present a similar skin surface contamination model in this region [17, 49]. Future experiments will address the possibility that insect vectors may be able to move *M*. *ulcerans* from one source and inject it into an animal or human.

Our focus on mechanical mosquito transmission arose from previous surveys in southeastern Australia where a strong association between *M*. *ulcerans* positive mosquitoes and human cases of BU has shown that *M*. *ulcerans* has not only been found on adult mosquitoes from both lab and field studies but also a biological gradient, where maximum likelihood estimates (MLE) of the proportion of *M*. *ulcerans*-positive mosquitoes increased as the number of cases of BU increased [22, 39, 50–53]. However, a recent study in Benin, West Africa found no evidence of *M*. *ulcerans* in association with adult mosquitoes [54]. The authors concluded that the mode of transmission might differ between southeastern Australia and Africa. Although, laboratory and fieldwork in West Africa suggest that aquatic insects, including mosquito larvae, play a role as reservoirs in nature for *M*. *ulcerans* that may be indirectly tied to transmission by serving as dispersal mechanisms [21, 26, 55]. Epidemiological studies have shown that direct contact with water is not a universal risk factor for BU [8, 11]. Prior exposure to insect bites and gardening are also independent risk factors for developing BU, while use of insect repellent is protective [11, 56].

Laboratory support to show mosquitoes can be competent vectors to spread BU is important additional evidence required to satisfy accepted vector ecology criteria for implicating insects in disease transmission [15, 37]. We found that infection was established following very minor penetrating trauma. Mosquitoes, in general, feed by insertion of a stylet, sheathed within the proboscis, beneath the skin of a host. The stylet has an approximate diameter 10 μM tapering to 1 μM at its tip and extending 1–2 mm below the skin surface. We estimated the density of *M*. *ulcerans* on the mouse-tails surface was 100–200 CFU/mm^2^. Thus, the number of bacteria potentially injected during mosquito feeding through this contaminated surface is likely to be low, but this is consistent with our infectious dose estimates from needle-stick punctures, indicating an ID50 of only 2.6 CFU (Fig 4B). *Aedes notoscriptus* mosquitoes are approximately twice as large as *Aedes aegypti*. Larger size may imply a longer stylet length, longer blood feeding time with a deeper penetration to a depth that may initiate *M*. *ulcerans* infection more frequently than that from *Aedes aegypti* mosquitoes that are smaller and do not blood feed as long. Such subtle differences in mosquito morphology and behavior may indicate why mechanical transmission in any form may be uncommon. There are strong parallels here with *Mycobacterium leprae*, the agent of leprosy. Like BU, the mode of transmission of the leprosy bacillus is unclear, but the infective dose is known to be very low (10 bacteria) and epidemiological evidence suggests multiple transmission pathways, including entry of the bacteria after skin trauma [57, 58]. Our infective dose estimate for *M*. *ulcerans* is consistent with observations that pathogens producing locally acting molecules to cause disease (e.g. the polyketide toxin mycolactone of *M*. *ulcerans*) have lower infective doses [59].

In summary, we have uncovered a highly efficient (88% rate) for needle stick skin punctures and a lower rate for mosquito punctures, suggesting a plausible mechanical transmission mode of *M*. *ulcerans* infection via anthropogenic or natural skin-puncturing microtrauma. We conclude from these experiments that reduction of exposure to insect bites, access to clean water for bathing, and prompt treatment of wounds and existing BU are concrete measures likely to interrupt BU transmission.

## Supporting information

S1 Table IS2404 mosquito qPCR results. (PDF)
