# Supplementary material for: Mycobacterium ulcerans low infectious dose and mechanical transmission support insect bites and puncturing injuries in the spread of Buruli ulcer
Source: PLoS Negl Trop Dis. 2017 Apr 14;11(4):e0005553. doi: 10.1371/journal.pntd.0005553 (PMC5406025; doi:10.1371/journal.pntd.0005553)

Supplementary Table S1: IS2404 mosquito qPCR results

Cycle threshold values (Ct) values	Mouse number	Number of mosquitoes biting	Legs			Head			Abdomen		
**Experiment 1**			**Avg**	**Std lower**	**Std upper**	**Avg**	**Std lower**	**Std upper**	**Avg**	**Std lower**	**Std upper**
A. notoscriptus - tail paint	182	1	22.74	-	-	27.923	-	-	27.673	-	-
	188	1	28.06	-	-	36.425	-	-	no ct	-	-
	189	1	32.373	-	-	no ct	-	-	no ct	-	-
	191	1	32.383	-	-	31.1	-	-	27.867	-	-
	192	1	no ct	-	-	no ct	-	-	no ct	-	-
	193	1	no ct	-	-	no ct	-	-	no ct	-	-
**Experiment 2**											
A. aegypti - tail paint	203	1	32.636	-	-	38.897	-	-	38.187	-	-
(multiple bites/tail so average Ct with SD summarised here)	204	4	35.093	34.025	36.161	35.53	32.809	38.251	38.117	36.073	40.161
	205	3	31.063	29.711	32.415	34.815	32.444	37.186	27.381	24.769	29.993
	206	2	30.948	30.376	31.52	33.785	29.348	33.692	33.825	28.11	39.54

Genome equivalents based on Ct values

	Mouse number	Number of mosquitoes biting	Legs Avg	Legs Std lower	Legs Std upper	Head Avg	Head Std lower	Head Std upper	Abdomen Avg	Abdomen Std lower	Abdomen Std upper
**Experiment 1**											
A. notoscriptus - tail paint	182	1	**6303.60**	-	-	**1120.14**	-	-	**1217.48**	-	-
	188	1	**1070.14**	-	-	**65.84**	-	-	**0.00**	-	-
	189	1	**254.14**	-	-	**0.00**	-	-	**0.00**	-	-
	191	1	**253.29**	-	-	**388.47**	-	-	**1141.25**	-	-
	192	1	0.00	-	-	**0.00**	-	-	**0.00**	-	-
	193	1	0.00	-	-	**0.00**	-	-	**0.00**	-	-
**Experiment 2**											
A. aegypti - tail paint	203	1	232.81	-	-	28.88	-	-	36.59	-	-
(multiple bites/tail so average Ct with SD summarised here)	204	4	102.64	146.53	71.90	88.73	219.76	35.82	37.46	74.04	18.95
	205	3	393.29	617.21	250.60	112.60	248.19	51.09	1341.95	3205.24	561.84
	206	2	408.66	494.50	337.72	158.73	696.60	163.73	156.63	1052.45	23.31

Conversion of Ct to genome equivalents

		Legs		Head		Abdomen
		**6303**		**1120**		**1217**
		**253**		**388**		**1141**
y=[-3.000Ln(x)+39.33]*25	**Average**	**3278**	**Average**	**754**	**Average**	**1179**
		1070		65		0
		254		0		0
		0		0		0
		0		0		0
	**Average E1**	**331**	**Average E1**	**16**	**Average E1**	**0**
		232		29		37
		103		89		38
		393		113		1342
		409		159		157
	**Average E2**	**284**	**Average E2**	**98**	**Average E2**	**394**

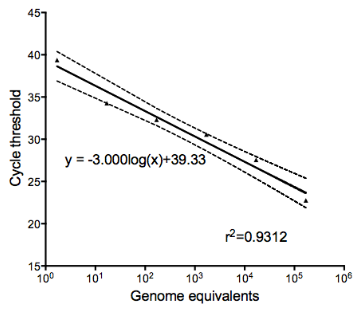

Supplement: S1 Table — (PDF) [file pntd.0005553.s001.pdf]
